# Prediction of autism spectrum disorder diagnosis using nonlinear measures of language-related EEG at 6 and 12 months

**DOI:** 10.1186/s11689-021-09405-x

**Published:** 2021-11-30

**Authors:** Fleming C. Peck, Laurel J. Gabard-Durnam, Carol L. Wilkinson, William Bosl, Helen Tager-Flusberg, Charles A. Nelson

**Affiliations:** 1 Division of Developmental Medicine, Boston Children’s Hospital, Harvard Medical School, Boston, MA 02115 USA; 2 Department of Neurology, Boston Children’s Hospital, Harvard Medical School, Boston, MA 02115 USA; 3 Princeton Neuroscience Institute, Princeton University, Princeton, NJ 08544 USA; 4 Department of Psychology, Northeastern University, Boston, MA 02118 USA; 5 Computational Health Informatics Program, Boston Children’s Hospital, Harvard Medical School, Boston, MA 02115 USA; 6 Health Informatics Program, University of San Francisco, San Francisco, CA 94117 USA; 7 Department of Psychological and Brain Sciences, Boston University, Boston, MA 02215 USA; 8 Harvard Graduate School of Education, Cambridge, MA 02138 USA

**Keywords:** EEG, Autism, Language development, Machine learning, Infant, Sensitive period

## Abstract

**Background:**

Early identification of autism spectrum disorder (ASD) provides an opportunity for early intervention and improved developmental outcomes. The use of electroencephalography (EEG) in infancy has shown promise in predicting later ASD diagnoses and in identifying neural mechanisms underlying the disorder. Given the high co-morbidity with language impairment, we and others have speculated that infants who are later diagnosed with ASD have altered language learning, including phoneme discrimination. Phoneme learning occurs rapidly in infancy, so altered neural substrates during the first year of life may serve as early, accurate indicators of later autism diagnosis.

**Methods:**

Using EEG data collected at two different ages during a passive phoneme task in infants with high familial risk for ASD, we compared the predictive accuracy of a combination of feature selection and machine learning models at 6 months (during native phoneme learning) and 12 months (after native phoneme learning), and we identified a single model with strong predictive accuracy (100%) for both ages. Samples at both ages were matched in size and diagnoses (*n* = 14 with later ASD; *n* = 40 without ASD). Features included a combination of power and nonlinear measures across the 10‑20 montage electrodes and 6 frequency bands. Predictive features at each age were compared both by feature characteristics and EEG scalp location. Additional prediction analyses were performed on all EEGs collected at 12 months; this larger sample included 67 HR infants (27 HR-ASD, 40 HR-noASD).

**Results:**

Using a combination of Pearson correlation feature selection and support vector machine classifier, 100% predictive diagnostic accuracy was observed at both 6 and 12 months. Predictive features differed between the models trained on 6- versus 12-month data. At 6 months, predictive features were biased to measures from central electrodes, power measures, and frequencies in the alpha range. At 12 months, predictive features were more distributed between power and nonlinear measures, and biased toward frequencies in the beta range. However, diagnosis prediction accuracy substantially decreased in the larger, more behaviorally heterogeneous 12-month sample.

**Conclusions:**

These results demonstrate that speech processing EEG measures can facilitate earlier identification of ASD but emphasize the need for age-specific predictive models with large sample sizes to develop clinically relevant classification algorithms.

**Supplementary Information:**

The online version contains supplementary material available at 10.1186/s11689-021-09405-x.

## Background

The past decade has witnessed a dramatic increase in the prevalence of autism spectrum disorder (ASD), a neurodevelopmental disorder characterized by deficits in social communication and repetitive and restrictive behaviors [1]. The CDC estimates that one in 54 children has an ASD diagnosis [2], up from the one in 88 prevalence reported about a decade ago [3]. Currently, ASD is diagnosed using behavioral measures, so a diagnosis cannot be made until toddlerhood or later when behavioral symptoms are reliably observable [4]. However, there is strong support for the assertion that early intervention leads to better intellectual and behavioral outcomes [5, 6]. Therefore, a central focus for the field has been to develop objective, biological markers to facilitate earlier detection, and subsequent intervention of ASD.

Neuroimaging measures provide strong candidate tools for early identification as they can be obtained from the newborn period onwards. For example, several recent studies have used magnetic resonance imaging (MRI) data collected in infancy to predict ASD diagnoses [7, 8]. However, MRI has several drawbacks, including expense and participant restrictions, making it a less feasible general screening tool. Electroencephalography (EEG), on the other hand, may prove to be a more scalable tool, given its low cost and ease of acquisition in awake and sleeping infants without participant restrictions. Moreover, EEG is known to be sensitive to brain-related changes in ASD before behavioral symptoms are observable [9–13]. Initial efforts to predict ASD diagnoses using baseline (i.e., resting-state) EEG early in life have shown promise [14–17]. However, diagnostic prediction using EEG recorded during tasks related to ASD symptoms has yet to be attempted and may outperform prior baseline EEG-based classification.

Language is frequently delayed or impaired in ASD [18–22], which may result from atypical peak synaptic sensitivity [23] and cortical excitatory and inhibitory imbalance [24] that disrupt neural circuits typically involved in language development (e.g., altered sensitive period dynamics). Therefore, focusing on the brain’s electrical activity during a language processing task may facilitate improved diagnostic prediction accuracy relative to baseline conditions, and provide insights into the neurobiology of language processing deficits within ASD. Notably, EEG has been used to measure differences in language processing in children with ASD who are older than 12 months [25–27], suggesting EEG is sensitive to atypical neural processing of language stimuli in ASD.

Perceptual narrowing of phoneme discrimination is a critical first stage in language acquisition [28]. Very young infants can discriminate between native and non-native phonemes better than adults, but they lose this ability over the first year of life as their phoneme perception is tuned to the language(s) experienced in daily life during this sensitive period of learning [29]. However, there is evidence that phoneme discrimination may develop differently in infants with ASD, thereby impacting language development [30–32]. This study focused on the phoneme learning sensitive period over the first year of life as a potential source of early indicators of subsequent ASD diagnosis.

There were two overarching goals of the present study. First, we aimed to evaluate whether EEG data collected during a language phoneme task at either 6 or 12 months of age in infants with familial risk for ASD can accurately predict later ASD diagnosis. We utilized EEG data collected from high familial risk infant siblings as part of a prospective longitudinal study, where diagnosis of ASD was determined at 2‑3 years of age. Though power analysis of EEG is most common, nonlinear measures can capture dynamical properties of the brain that power analysis is not able to quantify. For example, entropies evaluate the regularity and stability of patterns within the EEG signal, the fractal dimension measures self-similarity of a signal across multiple scales, and the Hurst exponent and detrended fluctuation analysis calculate long-term autocorrelation. Beyond capturing nonlinear patterns generated from a nonlinear system (i.e., the brain), these measures have exhibited sensitivity to changes in the brain’s balance of neural excitation and inhibition [33], and the ability to index transitions to epileptic seizures [34]. The excitatory-inhibitory balance of neural circuits undergoes critical developmental shifts during the first year of life with significant effects on neuroplasticity [35], including those posited to support language development, and each of these mechanisms are thought to be disrupted in ASD [32]. This prior evidence suggests that nonlinear dynamics are core features of healthy brain function and may relate to several fundamental neurodevelopmental processes over the first year of life. Nonlinear measures of adult EEG have accurately classified other clinical conditions, including depression [36–38], schizophrenia [39–41], and epilepsy [42–44]. Our lab previously found that these measures computed from resting-state EEG are useful in predicting ASD outcome [16], and we now aim to improve predictive capacity by evaluating these measures on language processing-related data. Second, given that expected perceptual narrowing of phoneme discrimination occurs between 6 and 12 months of age, we aimed to compare the EEG features most predictive of diagnosis and determine whether there are developmental differences in which features are most important during versus after the language phoneme learning period.

## Methods

### Study design and participant demographics

Participants were recruited to Boston Children’s Hospital to participate in a longitudinal study of infant siblings of children with ASD. Institutional review board approval was obtained from Boston University and Boston Children’s Hospital (# X06-08-0374). All infants had a gestational age greater than 36 weeks, no history of seizures, prenatal drug exposure, hearing impairment, or known genetic mutation involved in neurodevelopment. Infants were designated high risk (HR) for ASD based on the confirmed ASD clinical diagnosis of an older sibling. 104 HR infants were enrolled in the longitudinal study and ASD outcome was determined using the Autism Diagnostic Observation Schedule (ADOS) in conjunction with a clinical best estimate. For infants meeting criteria on the ADOS or coming within 3 points of clinical diagnosis cutoffs, a Licensed Clinical Psychologist reviewed scores and video recordings and provided a best estimate clinical judgment of ASD diagnosis.

Three sets of EEG data were evaluated in predictive models: a 6-month sample, full 12-month sample, and matching 12-month sample. At 6-months, EEGs from 54 HR infants were analyzed (14 HR-ASD, 40 HR-noASD). A “matching” 12-month dataset was curated to assess longitudinal changes in ASD prediction at the different ages. 13 HR-ASD and 24 HR-noASD participants contributed data at both timepoints. The single HR-ASD participant in the 6-month cohort who did not contribute data at 12 months was replaced by an HR-ASD, demographically matched participant. All 40 HR-noASD 12-month samples were included in the matched dataset, resulting in the same sample size as that of the 6-month dataset. Additional prediction analyses were performed on all EEGs collected at 12 months; this larger sample included 67 HR infants (27 HR-ASD, 40 HR-noASD). Demographic and data quality information of each outcome group is presented in Table 1. Fisher’s exact test was used to evaluate differences of demographic information between groups. The 12-month HR-ASD group had significantly lower mean maternal education than the HR-noASD group (*p* = 0.004). No other significant demographic differences were observed.

**Table 1:** Sample demographics of 6- and 12-month-old participants

	6-month dataset		12-month dataset		
	HR-ASD, ***n*** = 14	HR-noASD, ***n*** = 40	HR-ASD, ***n*** = 27	Matching HR-ASD, ***n*** = 14	HR-noASD, ***n*** = 40
Sex	8 M, 6 F	19 M, 21 F	17 M, 10 F	8 M, 6 F	18 M, 22 F
Child ethnicity (%)					
Caucasian	92.9	97.5	77.8	92.9	95
Hispanic/Latinx	21.4	2.5	14.8	21.4	7.5
Asian American	0	0	3.7	0	0
African American	0	0	3.7	0	0
Multirace	7.1	2.5	14.8	7.1	7.1
Mean household income ($1000s)	65‑75	65‑75	65‑75	65‑75	65‑75
Mean maternal education (%)					
< 4-year college	28.6	27.5	37	28.6	25
= 4-year college	35.7	15	37	42.9	12.5
> 4-year college	35.7	57.5	25.9	28.5	62.5
EEG HAPPE metrics (mean [SD])					
Length of raw EEG (s)	701.3 [212.9]	693.6 [220.7]	763.7 [213]	735.8 [225.2]	764.8 [209.5]
Good channels (%)	93.1 [4.1]	94.2 [4.7]	92 [4.8]	91.6 [4.7]	92.8 [5.5]
Rejected components (%)	47 [13]	44.1 [13]	43.8 [10]	42.6 [8.4]	43.4 [12.2]
EEG variance retained (%)	61.6 [13.3]	64.9 [17.4]	66.5 [12.6]	70.1 [10.5]	69.6 [16]
Mean retained artifact probability	0.16 [0.05]	0.17 [0.03]	0.15 [0.05]	0.16 [0.05]	0.15 [0.04]
Median retained artifact probability	0.13 [0.06]	0.15 [0.06]	0.1 [0.07]	0.11 [0.07]	0.1 [0.06]

### Behavioral assessments

The Mullen Scales for Early Learning (MSEL) and ADOS were administered at each data collection visit of the longitudinal study, including 6 and 12 months. The MSEL provides an index of ability in domains including language, cognition, and motor development.

### EEG paradigm

A subset of an oddball phoneme speech task was used for the present analysis, namely, only EEG data recorded during the standard (most frequent) English phoneme (a voiced, unaspirated dental/da/). Each trial consisted of the auditory stimulus played over 300 ms and followed by a variable interstimulus interval between 1000 and 1200 ms [31].

### EEG data acquisition and processing

EEG data were acquired in a dimly lit, sound-attenuated, electrically shielded room. A research assistant was present in the room to ensure that the infant remained calm and still during the language paradigm by blowing bubbles or presenting toys if the infant became distracted or fussy. Assistants did not engage in social interaction with the infant during task completion. EEG data were collected with either a 64-channel Geodesic Sensor Net or a 128-channel Hydrocel Geodesic Sensor Net (Electrical Geodesics, Inc. (EGI), Eugene, OR, USA), using a 0.1-Hz high-pass analog filter and online rereferencing to the vertex (channel Cz) through NetStation software (EGI, Eugene, OR, USA). Impedances were kept below 100 KΩ in accordance with the connected DC-coupled amplifier (Net Amps 200 or Net Amps 300, Electrical Geodesics, Inc.). Data were sampled at either 250 or 500 Hz.

EEG data were exported from NetStation to MATLAB format (R2017A). Files were batch processed using the Harvard Automated Processing Pipeline for EEG (HAPPE) within the Batch Electroencephalography Automated Processing Platform (BEAPP) software [45, 46].

**Fig. 1:**
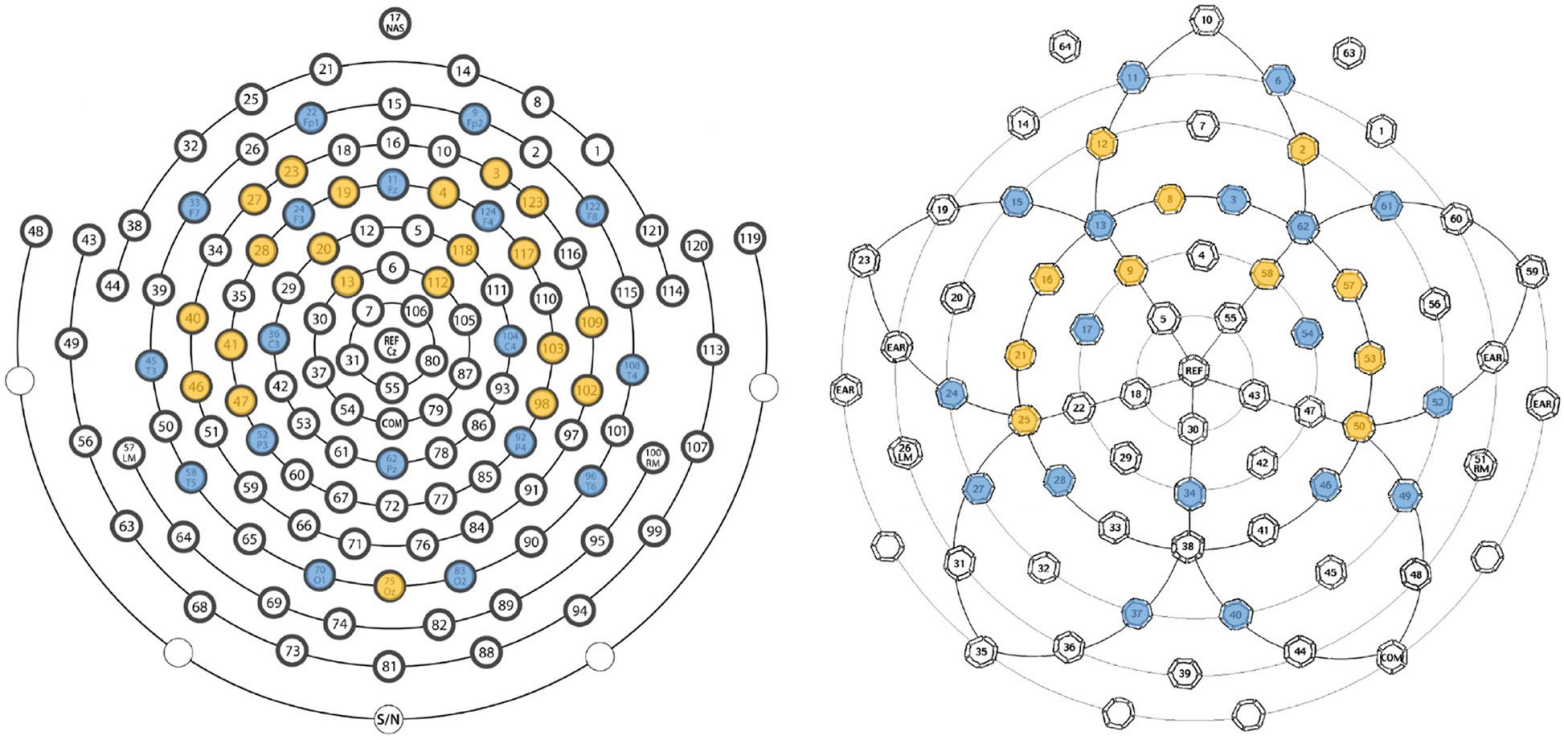
Two EEG nets were used in the study: the 128-channel EGI HydroCel Geodesic Sensor Net (version 1.0) presented on the left and the 64-channel EGI Geodesic Sensor Net (version 2.0) presented on the right. The 10-20 montage channels evaluated in this study are highlighted in blue, and HAPPE channels included in preprocessing steps are highlighted in yellow

Data were 1 Hz digital high-pass and 100 Hz low-pass filtered, downsampled to 250 Hz (if needed), and run through the HAPPE module using a spatially distributed subset of channels (Fig. 1). Default HAPPE artifact-rejection settings were used as they were optimized for this dataset prior to HAPPE’s original release. Namely, HAPPE artifact removal steps included bad channel identification, electrical line noise removal via Cleanline multitapering approach, artifact removal through wavelet-enhanced ICA and through a second ICA decomposition with automated component rejection above 50% artifact probability via the Multiple Artifact Rejection Algorithm [47, 48]. Bad channels were then interpolated and EEG data were re-referenced to the average reference and mean signal detrended.

### EEG data decomposition

The middle 20 s of the longest stretch of consecutive standard phoneme (English “da”) presentations in each file were selected for analysis to maximize the number of participants included while ensuring nonlinear measures could be calculated with fidelity. EEG data from 10‑20 montage channel equivalents (18 channels) for each net type were then decomposed into frequency sub-bands using a discrete wavelet transform and a coarse-graining procedure (see Supplementary Materials for description).

### EEG measures

Eleven different nonlinear measures and power were computed for each waveband from each of the 18 electrodes for each participant (Table 2).

**Table 2:** Descriptions of measures

	Abbreviation	Description
**Nonlinear variables**		
**NOLDS software package** [49]		
**Detrended fluctuation analysis**	DFA	Long-range correlation of the physiological time series
**Sample entropy**	SampE	Irregularity of physiological time series without self-matches
**Hurst exponent**	HurstE	Long-term memory processes of a time series
**Lyapunov exponent**	LyapE	Chaotic or periodic properties of a time series
**EntroPy (****https://github.com/raphaelvallat/entropy****)**		
**Permutation entropy**	PermE	Information content of a given time series based on probability distribution of a set of continuous points
**Spectral entropy**	SpecE	Degree of skewness in the frequency distribution
**Singular value decomposition entropy**	SVDE	Dimensionality of a time series.
**Approximate entropy**	AppE	Regularity of times series fluctuations
**Higuchi fractal dimension**	HFD	Self-similarity in time series using increasingly distanced samples in time
**Katz fractal dimension**	KFD	Complexity and self-similarity in time series using consecutive time points
**Lempel-Ziv complexity reference** [50]		
**Lempel Ziv complexity**	LZC	Randomness of finite sequences
**Linear variable**		
**SciPy signal processing** [51]		
**Power**	Power	Frequency amplitude of oscillations in a time series

### Feature selection strategies

Across models, three distinct feature selection methods were evaluated to prevent model overfitting: selection based on (1) the features most correlated with autism outcomes (Pearson correlation coefficient), (2) the features with most significant *F* ratio of mean square variances by group (*F* test), and (3) the features selected using recursive feature elimination (RFE) with cross validation based on a linear support vector machine. Each selection method was restricted to selecting 20 features.

### Classification strategies

We evaluated multiple prediction models that varied in classification approach, including a support vector machine (SVM) with radial basis function, a Gaussian Naïve Bayes algorithm, linear discriminant analysis, and a *k*-nearest neighbors model where *k* = 7, the square root of the sample size of the 6-month and matching 12-month datasets. All other models were trained using default parameters from the Python open source package *scikit-learn*.

Previous studies have found that including test samples in feature selection biases prediction accuracies [52], so we employed nested leave-one-out cross validation to evaluate the validity of model performance. Five metrics were used to evaluate model performance: accuracy, sensitivity, specificity, positive predictive value, and negative predictive value. Given the imbalanced nature of the sample, permutation testing was used to assess the significance of observed prediction accuracy. A null distribution of predictive accuracy was generated by repeating the following procedure over 1000 iterations: diagnostic labels were shuffled and cross validation analysis using features selected with true labels was performed.

### Characterizing selected features across 6 and 12 months

To determine the frequency of feature characteristics (i.e., channel, frequency band, measure) selected during the nested leave-one-out procedure (Fig. 1), the 20 features selected over each of the 54 iterations for 6-month and matching 12-month datasets were collated over the characteristic categories, summed, and then divided by the total iteration count. Significant differences between diagnostic groups were evaluated with a student’s *t* test with Bonferroni correction for multiple comparisons.

## Results

### Autism prediction at 6 and 12 months

Various prediction models drawing from three feature selection methods and four machine learning classifiers were evaluated for prediction accuracy of future autism diagnosis using either 6-month- or 12-month-matched EEG datasets (each with 40 HR-noASD and 14 HR-ASD participants). The SVM classifier with features selected by the Pearson correlation ranking method achieved 100% diagnostic prediction accuracy for both ages while other classification attempts were more variable (Table S2). Predictive accuracy using true labels with the SVM classifier was significantly better than chance as determined by the null distribution generated with random labels for both timepoints (6-month z-score 7.35, *p* < 0.0001; 12-month z-score 7.5, *p* < 0.0001).

### Predictive features at 6 and 12 months

The nature and spatial distribution of features selected in the successful 6- and 12-month predictive models were extracted in order to compare EEG features most predictive of ASD diagnosis either during or after perceptual narrowing of phoneme discrimination. Importantly, the same feature selection method and machine learning algorithm (Pearson correlation coefficient feature selection and an SVM classifier) achieved 100% predictive accuracy for both the 6-month- and 12-month-matched datasets, allowing for direct comparison of the features selected at each iteration of nested cross validation across the two ages.

**Fig. 2:**
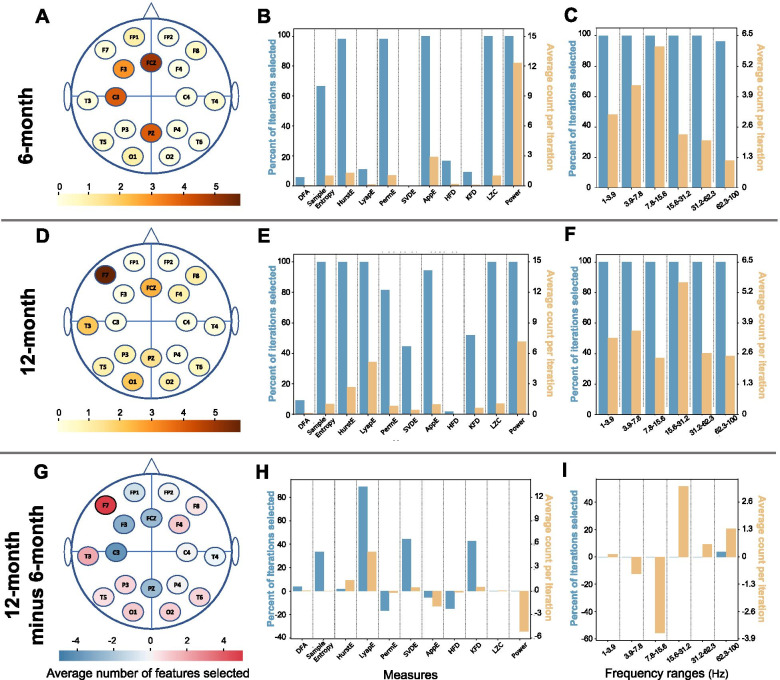
Information about features most correlated with autism diagnostic outcome for nearly overlapping 6- and 12-month analyses (*n* = 54). The bottom row visualizes the values for the 12-month dataset (middle row) minus the 6-month dataset (top row). **A**, **D**, **G** Average number of features selected from each channel. Color indicates number of features selected from a given channel. **B**, **E**, **H** Average count of each EEG measure across iterations (orange) and percentage of iterations that each measure was selected at least once (blue). **C**, **F**, **I** Average count of each wavelet across iterations (orange) and percentage of iterations that each wavelet was selected at least once (blue)

Figure 2 shows the selection rates of features by channel, measure, and frequency. At 6 months, features were selected largely from central and left of the midline locations (Fig. 2A), and power was the most frequently selected measure (Fig. 2B). Five of the 12 measures (power, approximate entropy, Hurst exponent, Lempel-Ziv complexity, and permutation entropy) were consistently selected across iterations. Within iterations, power was most frequently selected. Additionally, while almost all frequency bands were selected at each iteration, sub-bands with frequency ranges below 16 Hz were more frequently selected (Fig. 2C). At 12 months, selected channels changed most in the left hemisphere, with increased left lateralization (i.e., shifts away from midline) and representation from especially dense frontal and temporo-parietal scalp regions (Fig. 2D). Seven of the 12 measures were selected in at least 80% of the iterations, and half of the selected measures in each iteration were power or Lyapunov exponent computed at different wavebands and channels (Fig. 2E). While measures related to all frequency bands were consistently chosen across iterations, the average count per iteration of features related to the 15.6‑31.2 Hz range (largely canonical Beta frequencies) was nearly double at any other frequency range (Fig. 2F).

We next compared the mean value of each of the 20 most frequently selected features (see the “Methods” section: the “Characterizing selected features across 6 and 12 months” section) between ASD outcome groups (Table 3). After correcting for multiple comparisons, only approximate entropy computed from the F3 electrode in the delta range (1‑4 Hz) was significantly different between the two groups at 6 months (HR-ASD: mean 0.815 ± 0.08; HR-noASD: mean 0.76 ± 0.037; *p* < 0.0025). In contrast, at 12 months of age, the mean values measured for seven of the 20 features most commonly chosen across iterations were significantly different between groups such that HR-ASD infants had consistently higher values for each of these features than HR-noASD infants. Significant features across model iterations were also those that were most often chosen during feature selection (Fig. 2E): Lyapunov exponent, Hurst exponent, sample entropy, and power. Lempel-Ziv complexity was the only measure selected in all iterations that was not significantly different between groups after Bonferroni correction.

**Table 3:** Descriptions of the 20 most frequently selected features using the Pearson correlation coefficient features selection method. Scale in the frequency band feature category refers to the level of coarse-graining procedure (further described in the “Methods” section). Significance evaluated with paired sample *t* test corrected for 20 comparisons. Parameters that survived Bonferroni correction are in bold

	Feature			HR-ASD (mean ± std)	HR-noASD (mean ± std)	***p*** value	% Iterations selected
	Channel	Frequency	Measure				
6-month dataset	**F3**	**1‑3.9 Hz**	**AppE**	**0.815 ± 0.08**	**0.76 ± 0.037**	**< 0.0025**	**100**
	C3	1‑3.9 Hz	Power	0.038 ± 0.019	0.025 ± 0.012	< 0.05	100
	C3	7.8‑15.6 Hz	AppE	1.145 ± 0.035	1.115 ± 0.033	< 0.05	100
	Fz	15.6‑31.2 Hz	Power	0.018 ± 0.008	0.013 ± 0.006	< 0.05	100
	Fz	3.9‑7.8 Hz	Power	0.017 ± 0.007	0.012 ± 0.005	< 0.01	100
	Pz	15.6‑31.2 Hz	Power	0.029 ± 0.016	0.02 ± 0.009	< 0.01	100
	C3	3.9‑7.8 Hz	Power	0.026 ± 0.013	0.018 ± 0.009	< 0.05	98.1
	F3	7.8‑15.6 Hz	LZW	1.59 ± 0.027	1.563 ± 0.036	< 0.05	98.1
	Fz	1‑3.9 Hz	Power	0.032 ± 0.017	0.022 ± 0.01	< 0.05	98.1
	Fz	7.8‑15.6 Hz	Power	0.014 ± 0.007	0.01 ± 0.004	< 0.05	98.1
	Pz	31.2‑62.2 Hz	Power	0.028 ± 0.014	0.019 ± 0.009	< 0.01	98.1
	Pz	7.8‑15.6 Hz	Power	0.026 ± 0.017	0.017 ± 0.007	< 0.01	98.1
	C3	7.8‑15.6 Hz	Power	0.022 ± 0.01	0.016 ± 0.007	< 0.05	96.3
	F3	7.8‑15.6 Hz	PermE	2.575 ± 0.007	2.566 ± 0.012	< 0.05	96.3
	O1	62.5‑100 Hz	HurstE	0.137 ± 0.027	0.117 ± 0.025	< 0.05	92.6
	Pz	3.9‑7.8 Hz	Power	0.032 ± 0.016	0.023 ± 0.01	< 0.05	92.6
	Fz	31.2‑62.2 Hz	Power	0.018 ± 0.014	0.011 ± 0.006	< 0.05	90.7
	F8	3.9‑7.8 Hz	AppE	0.795 ± 0.038	0.763 ± 0.048	< 0.05	50
	FP1	1‑31.2 Hz (scale 3)	MSE	2.158 ± 0.058	2.11 ± 0.072	< 0.05	48.1
	FP1	1‑15.6 Hz (scale 4)	MSE	2.135 ± 0.085	2.084 ± 0.068	< 0.05	48.1
Matching 12-month dataset	**F7**	**7.8‑15.6 Hz**	**Power**	**0.031 ± 0.015**	**0.02 ± 0.008**	**< 0.001**	**100**
	**F7**	**3.9‑7.8 Hz**	**Power**	**0.044 ± 0.028**	**0.022 ± 0.011**	**< 0.001**	**100**
	**F7**	**1‑3.9 Hz**	**Power**	**0.07 ± 0.055**	**0.032 ± 0.015**	**< 0.001**	**100**
	**T3**	**1‑3.9 Hz**	**Power**	**0.107 ± 0.085**	**0.049 ± 0.033**	**< 0.001**	**100**
	**Pz**	**31.2‑62.2 Hz**	**HurstE**	**0.49 ± 0.06**	**0.418 ± 0.052**	**< 0.001**	**100**
	**F4**	**62.5‑100 Hz**	**LyapE**	**0.119 ± 0.009**	**0.112 ± 0.006**	**< 0.0025**	**100**
	**O1**	**15.6‑31.2 Hz**	**SampE**	**2.2 ± 0.038**	**2.139 ± 0.065**	**< 0.0025**	**100**
	F8	62.5‑100 Hz	LyapE	0.116 ± 0.007	0.111 ± 0.005	< 0.01	100
	Fz	15.6‑31.2 Hz	LZC	1.547 ± 0.021	1.53 ± 0.017	< 0.01	100
	F7	31.2‑62.2 Hz	Power	0.039 ± 0.028	0.023 ± 0.013	< 0.01	98.1
	F7	15.6‑31.2 Hz	Power	0.037 ± 0.019	0.024 ± 0.014	< 0.01	98.1
	Fz	7.8‑15.6 Hz	LyapE	0.049 ± 0.006	0.043 ± 0.008	< 0.01	98.1
	O2	3.9‑7.8 Hz	HurstE	0.563 ± 0.102	0.475 ± 0.098	< 0.01	98.1
	T3	3.9‑7.8 Hz	Power	0.064 ± 0.049	0.036 ± 0.024	< 0.01	98.1
	T5	1‑3.9 Hz	LyapE	0.04 ± 0.008	0.031 ± 0.011	< 0.01	98.1
	O1	15.6‑31.2 Hz	AppE	1.469 ± 0.029	1.448 ± 0.024	< 0.01	94.4
	P3	15.6‑31.2 Hz	LyapE	0.054 ± 0.005	0.049 ± 0.006	< 0.05	83.3
	F7	15.6‑31.2 Hz	PermE	2.583 ± 0.002	2.58 ± 0.004	< 0.05	81.5
	T6	3.9‑7.8 Hz	HurstE	0.539 ± 0.101	0.453 ± 0.115	< 0.05	53.7
	Pz	31.2‑62.2 Hz	SVDE	1.564 ± 0.017	1.546 ± 0.026	< 0.05	44.4

### Autism prediction at 12 months: full sample

We next evaluated the diagnosis prediction accuracy using all available 12-month participants and found a considerable decrease in almost all evaluation metrics (Table S2), including the combination of Pearson correlation coefficient feature selection and SVM classifier which dropped from 100 to 7% accuracy when the sample was expanded. Only one of the 12 classification schemes—*F* test feature selection with SVM algorithm—resulted in accuracy marginally above chance (61.2%) at the severe expense of sensitivity (3.7%), the measure evaluating the percentage of infants with ASD who were predicted correctly.

**Fig. 3:**
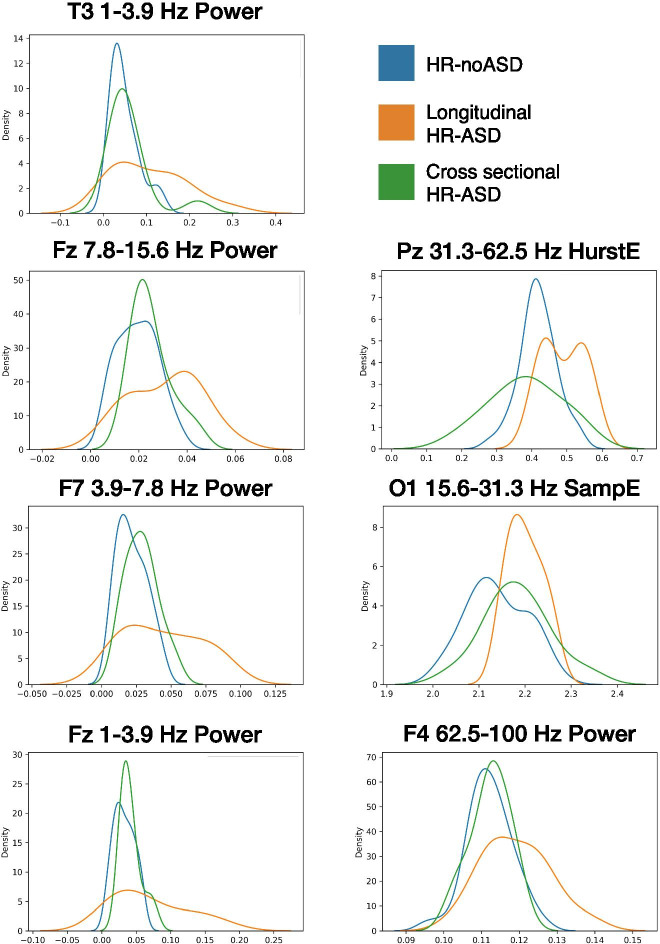
Feature distributions for features most significantly different between the longitudinal 12-month classification analyses (*n* = 54). Features are listed and emboldened in Table 3. Kernel density estimates are color coded by group: blue for HR-noASD (*n* = 40); orange for longitudinal HR-ASD (*n* = 14); and green for cross sectional HR-ASD (*n* = 13)

Given this discrepancy, we assessed whether there were behavioral differences in the HR-ASD participants between full and matched 12-month samples that could provide possible explanation for the differences in brain-based classification accuracy (Table 4). We found that 12-month-old participants who also participated at the 6-month timepoint had significantly lower severity scores on the 36-month ADOS compared to 12-month-old participants who only contributed data at the 12-month timepoint. Additionally, features that were significantly different between 12-month HR-noASD and longitudinal HR-ASD participants poorly discriminated between HR-noASD and cross sectional HR-ASD participants (Fig. 3).

**Table 4:** Behavioral assessments of 12-month HR-ASD group by participation timepoints. *P* value of *t* test comparing scores of each behavioral assessment between the matching and nonmatching 12-month HR-ASD infants (significant *p* value is emboldened). *ADOS* Autism Diagnostic Observation Schedule, *MSEL* Mullen Scales of Early Learning

	Full HR-ASD	Matching HR-ASD	Nonmatching HR-ASD	*p* value
Behavioral assessments (mean ± SD (*n*))				
ADOS				
24-month severity score	4.81 ± 2.5 (25)	4.21 ± 2.29 (14)	5.81 ± 2.79 (11)	0.15
36-month severity score	4.9 ± 2.19 (23)	3.85 ± 1.68 (13)	6.3 ± 2.93 (10)	**0.006**
12-month MSEL	*n* = 27	*n* = 14	*n* = 13	
Composite scaled score	99.0 ± 15.0	102.9 ± 13.3	94.8 ± 16.0	0.16
Verbal developmental quotient	90.0 ± 20.5	93.1 ± 20.4	86.7 ± 21.0	0.43
Non-verbal developmental quotient	116.7 ± 13.4	121 ± 12.1	112 ± 13.7	0.08

## Discussion

This study evaluated multiple classification schemes to predict ASD diagnosis in high-familial risk infants using language-task EEG data. Specifically, EEG was evaluated at 6 or 12 months of age, timepoints that span a critical early language-learning period in development. One hundred percent diagnostic classification accuracy was achieved using the Pearson correlation coefficient feature selection with the SVM classifier regardless of whether infants were within the critical period of language phoneme learning (6 months) or after (12 months). However, the features selected to achieve the 100% prediction rate differed between the two ages both in measure type and spatial distribution. Importantly, overall performance across models tested was highly variable and notably reduced when the sample demographics and size changed. Although we used robust statistical methods to limit overfitting in small samples, these constrained models may be unable to fit the variability present in ASD. The implications of these results in the search for early neuroimaging biomarkers of ASD diagnosis are discussed below.

### Developmental shift in predictive features

The longitudinal nature of the study provides the opportunity to assess whether predictive EEG features change across the course of a language-learning critical period over the first year of life. Several differences were identified. At 6 months, power from frequencies below 16 Hz dominated as the most common measures selected; in contrast, at 12 months, additional nonlinear measures were more consistently selected with a shift toward high frequencies, suggesting that nonlinear measures of high frequency signal better define binary ASD diagnostic outcomes based on language task EEG at 1 year of life. This trend is consistent with previous longitudinal studies that observe power differences between high- and low-risk infants emerging before 6 months but often dissipating by 12 months [9, 11, 14]. While power was the most common feature type selected at 6-months, few features were statistically different between HR-noASD and HR-ASD groups at this age after correcting for multiple comparisons. In contrast, at 12 months, significant differences between outcome groups were identified in 7 of the 20 most common features (Table 3). This suggests that different classification strategies are utilized at the two ages, with a combination of different power features taken together at 6 months and independent contributions of a range of power and nonlinear features at 12 months. McIntosh and colleagues previously used EEG to demonstrate that neural variability (measured with entropy) increases with neurodevelopment [53]. Therefore, the inclusion of more nonlinear measures versus power at 12 months compared to 6 months in the present study may indicate that these novel measures are more attuned to capturing variability inherent to development at the end of the first year of life, in line with previous research. Additionally, over the first year of life, the balance between excitation and inhibition in neural circuitry changes profoundly, so the sensitivity of entropy to excitation/inhibition dynamics [34] may advocate for their relevance in early-life development, consistent with the successful classification results dependent on entropy and other nonlinear measures.

We also observed differences between developmental time points topographically, with a shift leftwards from central-midline locations, including increased involvement of the temporal and lateral frontal scalp sites at 12 months (Fig. 2G). Power measures computed from the left-frontal F7 channel were particularly predictive as F7 power from all five frequency ranges was identified as 5 of the 20 most commonly selected features at 12 months (Table 3), and F7 power between 1 and 15.6 Hz was significantly increased in the HR-ASD group compared to HR-noASD. This shift may indicate that infants who are later diagnosed with ASD show atypical activity in the network of left-lateralized regions involved in typical language perception by 12 months of age [54–58]. Our EEG-related findings corroborate MRI findings of atypical activation related to passive auditory stimuli in adults and children with ASD over similar cortical areas [59, 60]. The window between 6 and 12 months is especially important for developing language ability, and the observed shift in the scalp sites included in successful prediction of ASD mirrors the developmental shifts in neural circuitry for language processing with development (e.g., left lateralization).

### Variable classification performance

Our evaluation of machine learning models identified a single model with high accuracy for matched 6- and 12-month datasets. However, the nested leave-one-out cross validation results varied greatly across different feature selection method and machine learning algorithm combinations. Performance variability may be attributed to the small size of the datasets, which risk overfitting or underfitting to the training data despite our efforts to minimize the dimensionality of the data before classification. Similarly sized studies using MRI data have previously demonstrated sensitivity lower than 90% with near-perfect specificity [7, 8]. These other studies only presented results from a single classification scheme, so the variability across classification schemes for MRI data is unknown.

The decrease in accuracy from 100% with the matched 12-month dataset to 7% with the full 12-month dataset using a SVM classifier suggests an inability to effectively separate the two diagnostic outcome classes after the HR-ASD group was expanded. Behavioral phenotypes of the HR-ASD infants added to complete the full 12-month analysis were more variable and severe than those who participated at both ages. It is possible that enrollment bias influenced the sample characteristics in that high-risk families enrolling at a later age may have had increased concerns about ASD related to observed symptoms. We postulate that the inclusion of more HR-ASD samples that had more similar feature distributions to the HR-noASD group resulted in increased feature overlap across outcome classes, preventing accurate hyperboundary separation (Fig. 3). It may also be that additional HR-ASD samples increased the heterogeneity of that group, essentially creating a continuum distribution of features across both groups. The result would be the same: A discriminating hyperboundary between groups would be difficult to find. Our 12-month results also suggest that more complex modeling will be required at this age to appropriately account for the full range of heterogeneity in ASD at the brain and behavioral levels. Simpler classification approaches might still perform well with an increased 6-month sample size if EEG measures tend to be less variable and better stratified between the ASD diagnostic outcomes at this younger age.

### The effect of heterogeneity and sample size on prediction

Autism is a heterogeneous disorder—its defining categories are broad and encompass a spectrum of symptom severity. Our observed decline in classification accuracy with increased sample size and important shifts in sample phenotypes (including ADOS severity) highlights factors that must be considered by the greater autism research community for future diagnosis prediction efforts. This within-study classification distinction serves as a case study of poor generalizability to a larger sample.

The HR-ASD infants who participated in both 6- and 12-month timepoints had significantly different ADOS severity scores at 36 months than the HR-ASD infants who joined the study at 12 months. On all five behavioral measures evaluated in Table 4, the 12-month-only HR-ASD group had higher ADOS scores and lower MSEL scores (corresponding to overall lower indexes of development). We hypothesize that the inclusion of a more severe and variable dataset reduced the accuracy of classification since we were predicting ASD as a binary diagnosis. Using resting-state EEG from the same infant-sibling dataset in this analysis, we have previously observed a similar lower accuracy in a 12-month sample, although a similar size sample at 9 months had high accuracy. As discussed in our previous paper, this may be a real neurodevelopmental trend reflecting the cross-over of neurodevelopmental trajectories of infants who do and do not go on to develop ASD [15, 16]. Alternatively, this finding may correspond with previous meta-analyses of brain-disorder prediction field that have found decreasing accuracies reported as sample sizes—and, importantly, heterogeneity within the sample—increase [61–63]. Overall, more sample data are needed in order to more completely represent the brain activity differences that arise in ASD, which would also permit the use of more complex models that may more appropriately account for the variability and complex associations between brain activity and diagnosis.

### Limitations and future directions

We acknowledge several limitations of the current study in addition to the discussed challenges of sample size and heterogeneity. First, our focus on infants with familial risk of autism may not generalize to other ASD-risk groups or to the general population. Second, the specificity of our findings to ASD (versus other comorbid conditions) is unknown. Further testing across clinical populations (e.g., global developmental delay without ASD or isolated language delay) is needed to understand whether EEG could also be used to predict comorbidities with significant impact on functional outcomes. This study determines ASD outcome at age 3, which is appropriate for assessing ASD but not for many other developmental conditions that emerge across early childhood. Therefore, questions about comorbidity call for the extension of longitudinal studies to track participants beyond 3 years to capture a more complete clinical description of participants. Third, our sample was not diverse ethnically, racially, or in income level. Predictive analyses require not only large sample sizes but also must include infants from diverse populations in order to improve clinical applicability to the general population. These results suggest that collaboration across samples is critical to moving forward in developing early predictive models.

Future studies of early predictive markers of ASD and other neurodevelopmental disorders need to be acutely aware of participant age, given the dramatic developmental changes in predictive feature profiles over the 6-month age window in the study. Moreover, given the variability of behavioral measures within the ASD outcome group, future studies should consider distinguishing different subpopulations of ASD grouped by biological presentations or phenotype profiles at the behavioral level as opposed to only binary diagnosis.

## Conclusions

These results demonstrate that speech processing EEG measures may facilitate earlier identification of ASD. However, different nonlinear and power measures were predictive of ASD outcomes depending on developmental age with respect to early language learning. Overall, these findings emphasize the need for age-specific predictive models with large sample sizes and the challenge of discriminating diagnostic differences in highly heterogeneous populations.

## Supplementary Information

**Additional file 1: Supplemental Information.** Provides additional descriptions of (1) Wavelet decomposition and coarse-graining procedure, (2) Classification strategies. Supplemental Table provides comprehensive evaluation metrics for machine learning classification results the three data sets.

## Data Availability

Python and Jupyter Notebook scripts used for computation of nonlinear measures and machine learning analysis are available. The datasets of preprocessed nonlinear and power measures analyzed during the current study are available from the corresponding author on reasonable request.

## Abbreviations

ADOS: Autism Diagnostic Observation Schedule
ASD: Autism spectrum disorder
BEAPP: Batch EEG automated processing platform
EEG: Electroencephalography
EGI: Electrical Geodesics, Inc
HAPPE: Harvard automated preprocessing pipeline for EEG
HR: High risk
MARA: Multiple Artifact Rejection Algorithm
MRI: Magnetic resonance imaging
MSEL: Mullen Scales of Early Learning
SVM: Support vector machine
