# Supplementary material for: Prediction of autism spectrum disorder diagnosis using nonlinear measures of language-related EEG at 6 and 12 months
Source: J Neurodev Disord. 2021 Nov 30;13:57. doi: 10.1186/s11689-021-09405-x (PMC8903497; doi:10.1186/s11689-021-09405-x)
Supplement: Supplementary file 1 — Additional file 1: Supplemental Information. Provides additional descriptions of (1) Wavelet decomposition and coarse-graining procedure, (2) Classification strategies. Supplemental Table provides comprehensive evaluation metrics for machine learning classification results the three data sets. [file 11689_2021_9405_MOESM1_ESM.pdf]

Supplemental Information

Wavelet decomposition and coarse-graining procedure

Discrete wavelet transform localizes a signal in both the time and frequency domains, and it is a useful tool that allows analysis of narrow frequency sub-bands of the original signal thought to have biological relevance. The discrete wavelet transform is a filter that decomposes the signal into high pass (details) and low pass (approximations) sub-bands, which are down-sampled to half of the sampling rate. Note that an upper and lower bound of 100 Hz and 1 Hz, respectively, were imposed on the signal in the preprocessing signal steps before wavelet decomposition. The discrete wavelet transform was performed using the PyWavelet *dwt* function with the Daubechies 4 wavelet and symmetric signal extension on the approximate signal of the previous level of decomposition, starting with the original signal at Level 0. Table 1 lists the wavebands that correspond to frequency ranges of wavebands used in traditional EEG power spectral analyses.

TABLE S1

Wavelet level	Wavelet frequency range	Approximate EEG waveband
**D1**	62.5 - 100 Hz	high gamma
**D2**	31.3 - 62.5 Hz	low gamma
**D3**	15.6 - 31.3 Hz	beta
**D4**	7.8 - 15.6 Hz	alpha
**D5**	3.9 - 7.8 Hz	theta
**A5**	1-3.9 Hz	delta

Table S1. Wavelet decomposition frequency bands for wavelet levels associated with traditional EEG wavebands.

Only these non-overlapping frequency bands resulting from the wavelet decomposition–D1, D2, D3, D4, D5, and A5–were selected for further analysis with nonlinear measures and covered the range from 1-100 Hz.

In addition to the wavelet decomposition, a coarse-grained time series was computed from the original signal using the multiscale entropy analysis methodology introduced by Costa and colleagues (5). The coarse-graining procedure involves dividing the time series into

nonoverlapping windows of size *t* and averaging data points within each window, resulting in a signal that is 1/*t* times the length of the original signal. Each scale corresponds to window size: the second scale coarse grains with *t* of two, the third with three, and so on. Five scales were computed from the signal of each electrode, and only multiscale entropy was computed from each of the sub-signals. We note that the coarse-graining procedure is equivalent to using the overlapping frequency bands obtained from the approximations in a discrete wavelet transform using a Haar basis (6).

Classification strategies

There are two main foundational types of supervised learning models, discriminative and generative, that predict the conditional probability of an outcome given input features. Generative models model the distribution of the individual classes with a joint probability distribution. Discriminative models, on the other hand, learn explicit boundaries that best separate the given classes with the conditional probability distribution. We evaluated two generative classifiers, the Naïve Bayes algorithm and linear discriminant analysis, and one discriminative model, an SVM with radial basis functions. Additionally, we included k-nearest neighbors, which is considered a discriminative classifier as well but really employs a voting method based on the outcomes of the *k* nearest neighbors in feature space using a simple Euclidean distance metric. The number of neighbors was set to 7, the square root of the sample size of the 6-month and matching 12-month datasets, and we maintained a selection of 7 neighbors for the full 12-month dataset for consistency. All feature selection methods and machine learning algorithms were used for each dataset analysis, resulting in evaluation of 12 different classification schemes.

References

1. NITRC: CleanLine: Tool/Resource Info (n.d.): Retrieved February 6, 2021, from https://www.nitrc.org/projects/cleanline
2. Winkler I, Brandl S, Horn F, Waldburger E, Allefeld C, Tangermann M (2014): Robust artifactual independent component classification for BCI practitioners. *Journal of Neural Engineering* 11. https://doi.org/10.1088/1741-2560/11/3/035013

3. Levin AR, Leal ASM, Gabard-Durnam LJ, O'Leary HM (2018): BEAPP: The batch electroencephalography automated processing platform. *Frontiers in Neuroscience* 12. https://doi.org/10.3389/fnins.2018.00513
4. Gabard-Durnam LJ, Mendez Leal AS, Wilkinson CL, Levin AR (2018): The Harvard Automated Processing Pipeline for Electroencephalography (HAPPE): Standardized Processing Software for Developmental and High-Artifact Data. *Frontiers in Neuroscience* 12: 97.
5. Costa M, Goldberger AL, Peng CK (2005): Multiscale entropy analysis of biological signals. *Physical Review E - Statistical, Nonlinear, and Soft Matter Physics* 71. https://doi.org/10.1103/PhysRevE.71.021906
6. Bosl WJ, Tager-Flusberg H, Nelson CA (2018): EEG Analytics for Early Detection of Autism Spectrum Disorder: A data-driven approach. *Scientific Reports* 8. https://doi.org/10.1038/s41598-018-24318-x

Table S2

Feature selection method		Pearson correlation coefficient				F-test				Recursive feature elimination			
Machine learning algorithm		SVM	KNN	LDA	NB	SVM	KNN	LDA	NB	SVM	KNN	LDA	NB
Dataset	Evaluation metrics (%)												
6 month	PPV	100	0	27.3	50	23.1	55.6	21.6	83.3	22.2	66.7	13	30.3
	NPV	100	73.6	74.4	75	0	80	0	81.3	72.2	76.5	64.5	81
	SENS	100	0	21.4	7.1	85.7	35.7	78.6	35.7	28.6	14.3	21.4	71.4
	SPEC	100	97.5	80	97.5	0	90	0	97.5	65	97.5	50	42.5
	ACC	100	72.2	64.8	74.1	22.2	75.9	20.4	81.5	55.6	75.9	42.6	50
Matching 12 month	PPV	100	60	29.4	57.1	43.8	50	21.7	24.5	23.5	27.3	25	31
	NPV	100	77.6	75.7	78.7	81.6	75	50	60	73	74.4	73.3	80
	SENS	100	21.4	35.7	28.6	50	7.1	71.4	85.7	28.6	21.4	42.9	64.3
	SPEC	100	95	70	92.5	77.5	97.5	10	7.5	67.5	80	55	50
	ACC	100	75.9	61.1	75.9	70.4	74.1	25.9	27.8	57.4	64.8	51.9	53.7
Full 12 month	PPV	11.1	41.7	15.4	9.1	100	47.1	33.3	36.8	12.8	23.5	35.5	31.3
	NPV	0	60.5	43.9	44.4	60.6	62	57.7	58.3	21.4	42.4	55.6	51.4
	SENS	18.5	37	14.8	7.4	3.7	29.6	18.5	25.9	18.5	29.6	40.7	37
	SPEC	0	65	45	50	100	77.5	75	70	15	35	50	45
	ACC	7.5	53.7	32.8	32.8	61.2	58.2	52.2	52.2	16.4	32.8	46.3	41.8

Table S2: Evaluation metrics for machine learning classification results reported in percentages. All three datasets are reported: 6-month dataset (n = 54; 74.1% HR-noASD), matching 12-month dataset (n = 54; 74.1% HR-noASD), and full 12-month dataset (n = 67; 59.7% HR-noASD). Machine learning classification was evaluated in terms of positive predictive value (PPV), negative predictive value (NPV), sensitivity (SENS), specificity (SPEC), and accuracy (ACC). Evaluation metrics are included for four algorithms: nonlinear support vector machine (SVM), K-nearest neighbors with 7 neighbors (KNN), linear discriminant analysis (LDA), and Naïve Bayes (NB). 20 features were selected by the noted selection methods before nested leave-one-out cross validation using each of the listed machine learning algorithm.
